# Cortical and Subcortical Structural Abnormalities in Patients With Idiopathic Cervical and Generalized Dystonia

**DOI:** 10.3389/fnimg.2022.807850

**Published:** 2022-03-31

**Authors:** Yunhao Wu, Tao Wang, Qiong Ding, Hongxia Li, Yiwen Wu, Dianyou Li, Bomin Sun, Yixin Pan

**Affiliations:** ^1^Department of Neurosurgery, Center for Functional Neurosurgery, Ruijin Hospital, Shanghai Jiao Tong University School of Medicine, Shanghai, China; ^2^Institute of Science and Technology for Brain-Inspired Intelligence, Fudan University, Shanghai, China; ^3^Department of Neurology, Institute of Neurology, Ruijin Hospital, Shanghai Jiao Tong University School of Medicine, Shanghai, China

**Keywords:** cervical dystonia, generalized dystonia, cortical thickness, volumetric analysis, shape analysis

## Abstract

**Objectives:**

In this study, we sought to investigate structural imaging alterations of patients with idiopathic dystonia at the cortical and subcortical levels. The common and specific changes in two subtypes of dystonia, cervical dystonia (CD) and generalized dystonia (GD), were intended to be explored. Additionally, we sought to identify the morphometric measurements which might be related to patients' clinical characteristics, thus providing more clues of specific brain regions involved in the mechanism of idiopathic dystonia.

**Methods:**

3D T1-weighted MRI scans were acquired from 56 patients with idiopathic dystonia and 30 healthy controls (HC). Patients were classified as CD or GD, according to the distinct symptom distributions. Cortical thickness (CT) of 30 CD and 26 GD were estimated and compared to HCs using Computational Anatomy Toolbox (CAT12), while volumes of subcortical structures and their shape alterations (29 CD, 25 GD, and 27 HCs) were analyzed *via* FSL software. Further, we applied correlation analyses between the above imaging measurements with significant differences and patients' clinical characteristics.

**Results:**

The results of comparisons between the two patient groups and HCs were highly consistent, demonstrating increased CT of bilateral postcentral, superiorparietal, superiorfrontal/rostralmiddlefrontal, occipital gyrus, etc., and decreased CT of bilateral cingulate, insula, entorhinal, and fusiform gyrus (*P*_FWE_ < 0.005 at the cluster level). In CD, trends of negative correlations were found between disease severity and CT alterations mostly located in pre/postcentral, rostralmiddlefrontal, superiorparietal, and supramarginal regions. Besides, volumes of bilateral putamen, caudate, and thalamus were significantly reduced in both patient groups, while pallidum volume reduction was also presented in GD compared to HCs. Caudate volume reduction had a trend of correlation to increasing disease severity in GD. Last, shape analysis directly demonstrated regional surface alterations in bilateral thalamus and caudate, where the atrophy located in the head of caudate had a trend of correlation to earlier ages of onset in GD.

**Conclusions:**

Our study demonstrates wide-spread morphometric changes of CT, subcortical volumes, and shapes in idiopathic dystonia. CD and GD presented similar patterns of morphometric abnormalities, indicating shared underlying mechanisms in two different disease forms. Especially, the clinical associations of CT of multiple brain regions with disease severity, and altered volume/shape of caudate with disease severity/age of onset separately in CD and GD might serve as potential biomarkers for further disease exploration.

## Introduction

Dystonia is a rare neurological disease characterized by involuntary muscle contractions leading to abnormal postures and movements. Classification of the disease based on distinct symptom distribution is clinically important and widely used for different implications of therapy (Albanese et al., 2013). Cervical dystonia (CD) and generalized dystonia (GD) are two common subtypes. While the premier subtype is a typical focal form involving exclusive neck twisting/abnormal posture, the latter mainly affects the trunk and even wider body regions. Since idiopathic dystonia begins without certain causes or positive findings in conventional imaging scans, itis challenging to trace the abnormal brain structures or circuits involved in the mechanism of the disease.

The pathophysiological landscape of dystonia has been updated in the past few years, helping us get a clearer insight into its mechanism. Initially, the knowledge that injuries of certain brain nuclei (basal ganglia and thalamus most frequently) could lead to secondary dystonia, making those structures serve as causative roles in the disease. For decades, brain circuit alterations in idiopathic dystonia have been attested in several cortical and subcortical brain regions (Comella, 2018; Okromelidze et al., 2020), which updated the perspectives that the disease arises from network dysfunction rather than a single structure. Currently, theories of intracortical inhibition loss, sensory dysfunction, and homeostatic plasticity disruption have been proposed as three main changes in dystonia (Quartarone and Hallett, 2013). Meanwhile, neuroimaging findings of structural, functional, and metabolic abnormalities mainly indicated disrupted basal the ganglia–thalamo–cortical, cerebello–thalamo—cortical circuit pathways, etc. (Zoons et al., 2011; Karimi and Perlmutter, 2015).

To explore structural abnormalities in dystonia, voxel-based morphometry (VBM) and diffusion-tensor imaging (DTI) were commonly used methods for measuring gray matter volume (GMV) (Whitwell and Josephs, 2007) and white fiber integrity changes, respectively (Mori and Zhang, 2006). Observed decreased or increased GMV in patients with focal dystonia had been concentratedly reported in the basal ganglia, thalamus, primary sensorimotor and associate areas (frontal, temporal, occipital gyrus, etc.), which are responsible for motor selection, sensorimotor integration, movement imagination, and even higher-order cognitive functions (Egger et al., 2007; Obermann et al., 2007). In DTI studies, abnormal fractional anisotropy (FA) were consistently detected in pathways to and from the basal ganglia, thalamus and the sensorimotor projections, implying impaired white matter integrities connecting those regions (Bonilha et al., 2007; Yang et al., 2014).

In addition, more accurate and complement imaging techniques have been enabled to provide supplementary information of the brain morphometric changes in dystonia currently. For example, surface-based morphometry (SBM) possesses several advantages over VBM, particularly in allowing the estimation of cortical thickness (CT) and gyrification indices (Desai et al., 2005), which is more suitable for describing cortical column abnormalities. Meanwhile, subcortical structures including the thalamus and basal ganglia (BG) can be precisely segmented and estimated for shape analysis. Up to now, five studies (Simonyan and Ludlow, 2012; Cerasa et al., 2014; Bianchi et al., 2017; Vilany et al., 2017; Tomić et al., 2021) have investigated CT alterations in different forms of idiopathic dystonia, embracing focal, task-specific/non-task-specific dystonia, and dystonia combined with tremor. Vilany et al. demonstrated cortical atrophy in motor, sensory, visual, and limbic regions in craniocervical dystonia patients compared to healthy controls (HC) (Vilany et al., 2017). In another study targeting spasmodic dysphonia, however, increased CT was presented in the laryngeal sensorimotor cortex, inferior frontal gyrus, superior/middle temporal, and supramarginal gyrus (Simonyan and Ludlow, 2012). For the heterogeneity of the disease and the lack of comprehensive imaging evaluations, more evidence of cortical and subcortical morphometric measurements in distinct dystonia subtypes is necessary.

Here, we used computational anatomy toolbox (CAT12) and FSL software to investigate CT, and volumes and shapes of subcortical structures in two subtypes of idiopathic dystonia. The results may strengthen the current literature and provide supplementary imaging information of the disease.

First, the purpose of this study was to explore the structural alterations of idiopathic dystonia from different fields. According to preliminary evidence from previous VBM or DTI studies, we hypothesized that the abnormalities would not be restrained to solitary structure, and regions responsible for sensorimotor integration and higher-order cognitive controlling networks might be largely included. In addition, we intended to explore the common and specific changes in CD and GD, implying their shared and individual mechanisms. Finally, correlation analyses were performed between the imaging measurements with significant differences and clinical characteristics of patients (age at onset, disease duration, rating scores reflecting motor, disability severity, etc.) to find out potential biomarkers affected by the disease.

## Materials and Methods

### Subjects

This retrospective study was approved by the local Research Ethics Committee and informed written consents were obtained from all participants. Fifty-six patients with dystonia and thirty HC were enrolled in this study from 2015 to 2020, and there were no statistical age or gender differences among the three groups (Table 1). All participants were right-handed. Patients were diagnosed as idiopathic CD (30 of 56) or GD (26 of 56) by experienced neurologists. The necks were exclusively affected in patients with CD, while twisting/abnormal posture of the trunk and one extremity involved at least were presented in patients with GD. Disease severity ratings were assessed using Toronto Western Spasmodic Torticollis Rating Scale (TWSTRS) for CD and Burke-Fahn-Marsden Dystonia Rating Scale (BFMDRS) for GD. All patients received regular botulinum toxin injections or oral medications to manage their symptoms. To be fully symptomatic at the time of assessment and scanning, patients had at least a 3-month interval since their last botulinum toxin injection and a 12-h interval since the last oral medication.

**Table 1:** Demographic data of patients with dystonia and healthy controls.

	**CD (*n* = 30)**	**GD (*n* = 26)**	**HC (*n* = 30)**	***P*-value**
Age (years)	45.70 ± 13.79	44.46 ± 18.30	38.73 ± 11.26	0.136
Gender (male/female)	15/15	15/11	19/11	0.578
Age of onset (years)	41.67 ± 13.41	35.69 ± 20.95	–	0.203
Disease duration (years)	4.12 ± 5.92	8.85 ± 10.21	–	0.036
**TWSTRS**
Total scores	39.64 ± 12.58	–	–	–
Severity scores	17.07 ± 4.77	–	–	–
Disability scores	18.03 ± 4.47	–	–	–
Pain scores	4.88 ± 3.66	–	–	–
**BFMDRS**
Total scores	–	43.19 ± 20.14	–	–
Motor scores	–	31.15 ± 15.25	–	–
Disability scores	–	11.65 ± 5.59	–	–

*Values are means ± standard deviation for continuous variables and percentage for categorical variables (gender), respectively. P-values of age in three groups are from one-way analysis of variance (ANOVA) models, and P-values of gender are from Pearson Chi-Square. P-values of other clinical variables' comparisons between two patient groups are from independent samples t-test. CD, cervical dystonia; GD, generalized dystonia. TWSTRS, Toronto Western Spasmodic Torticollis Rating Scale; BFMDRS, Burke-Fahn-Marsden Dystonia Rating Scale*.

The exclusion criteria included: (a) age under 18 years; (b) any histories of brain surgeries or combined neurological/psychiatric conditions; (c) confirmed hereditary or acquired pathogenic factors, including injury, anoxia, cerebral vascular disease, etc.; and (d) obvious brain structural abnormalities found in conventional MRI scans (T1and T2 weighted images).

### Data Acquisition

MRI data were acquired using a 3.0 T MRI system and an eight-channel phase array head coil (GE Healthcare Signa HDx 3.0 T, Piscataway, NJ, USA). A high-resolution T1-weighted 3D spoiled gradient recall sequence was used (TR = 7.0 ms, TE = 3.0 ms, flip angle = 7, inversion time = 936 ms, slice thickness = 1.0 mm, field of view = 256 × 256 mm^2^, spatial resolution = 1 × 1 × 1 mm^3^). Image data with poor quality and the absence of significant brain pathology or artifacts were excluded.

### Cortical Thickness Estimation

We applied the SBM method for CT estimation. All participants' data were processed with the CAT12 toolbox (http://www.neuro.uni-jena.de/cat/; version 1700) within SPM12 (http://www.fil.ion.ucl.ac.uk/spm/software/spm12) and MATLAB R2020a (Mathworks, Natick, Massachusetts). Initially, all volumes were automatically segmented, where CT estimation and central cortical surface creation for each hemisphere were performed using the projection-based thickness (PBT) method (Dahnke et al., 2013). Topological correction (Yotter et al., 2011a), spherical mapping (Yotter et al., 2011b), and spherical registration (Ashburner, 2007) were subsequently carried out within one step. The output thickness data was resampled and smoothed in a subsequent step, applying 15 mm FWHM smoothing kernels.

Additionally, we extracted CT values of regions of interest (ROIs) from each individual surface, based on Desikan–Killiany Atlas (DK40) (Desikan et al., 2006). Mean values inside the referred ROIs were applied for correlation analysis.

### Volumetric and Shape Estimation of Subcortical Regions

Volumetric and shape analysis of subcortical regions were implemented in FSL software (version 6.0.0, https://fsl.fmrib.ox.ac.uk/). Bilateral thalamus and BG (pallidum, putamen, and caudate) were selected to be analyzed in this study. We performed nuclei segmentation using FIRST (Patenaude et al., 2011), a model-based segmentation and registration tool, which can automatically parameterize volumetric labels in terms of meshes. The training models were constructed from manually segmented images of 336 subjects, provided by the Center for Morphometric Analysis.

The quality of segmentation of each subject was checked by two radiologists independently, with low-quality results and outlier volumetric values eliminated (the outlier values were defined as values beyond the range of average value ± triple standard deviation, then one CD patient, one GD patient, and three HC patients were excluded). 29CD, 25GD, and 27HC were included in the final volumetric and shape analysis. Fslstats module in FSL was used to measure the volumes of segmented and boundary-corrected subcortical structures. First, utils was used to process the vertex location projection values of each subject, referring to the cohort average shape.

### Statistical Analyses

Cortical thickness were compared among three groups using independent 2-sample *t*-test within the general linear model (GLM) in SPM12. Comparisons of subcortical volumes were performed in SPSS 15 software. We considered age and gender as nuisance variables, and the total intracranial volume (TIV) was additionally adjusted in volumetric analyses. CT results are considered significant at *P* < 0.005 at the cluster level (3-group comparisons for each hemisphere, 0.005 <0.05/6), corrected for multiple comparisons with family wise error (FWE). This threshold (*P* < 0.005) was also employed in subcortical volume and shape comparisons (3-group comparisons for subcortical nucleus of each hemisphere). Further, DK40 ROIs were selected according to the results from vertex-wise statistics, which overlapped main significant clusters. Values of patients' CT of ROIs and subcortical volumes were extracted and separately correlated to clinical characteristics including age at onset, disease duration, TWSTRS severity/BFMDRS motor scores, TWSTRS/BFMDRS disability scores, and TWSTRS pain scores in each group. Age, gender, and TIV (only in volumetric analyses) were adjusted.

We calculated vertex-wise statistics to investigate regional shape alterations of subcortical structures between two groups that had demonstrated total volumetric differences in the previous step. Partial correlation analyses (age, gender-adjusted) were subsequently performed to identify associations of subcortical shape differences with patients' clinical characteristics that have been mentioned above.GLM and permutation testing using Randomize module in FSL were applied for statistics calculation, with the threshold-free cluster enhancement (TFCE) identifying clusters of voxels with significance (permutation times: *n*= 5000) (Smith and Nichols, 2009).

In all correlation analyses, however, we employed a threshold of *P* < 0.0025 for multiple testing corrections. MRI metrics of each hemisphere in the two patient groups were separately correlated to clinical characteristics (age at onset, disease duration, TWSTRS severity/BFMDRS motor scores, TWSTRS/BFMDRS disability scores, and TWSTRS pain scores), thus leading to 20 (2^*^2^*^5) tests.

As the corrected threshold levels were very strict in our analyses, and uncorrected threshold of *P* < 0.05 was considered as trend-level for exploratory purposes.

## Results

### Cortical Thickness

**Figure 1:**
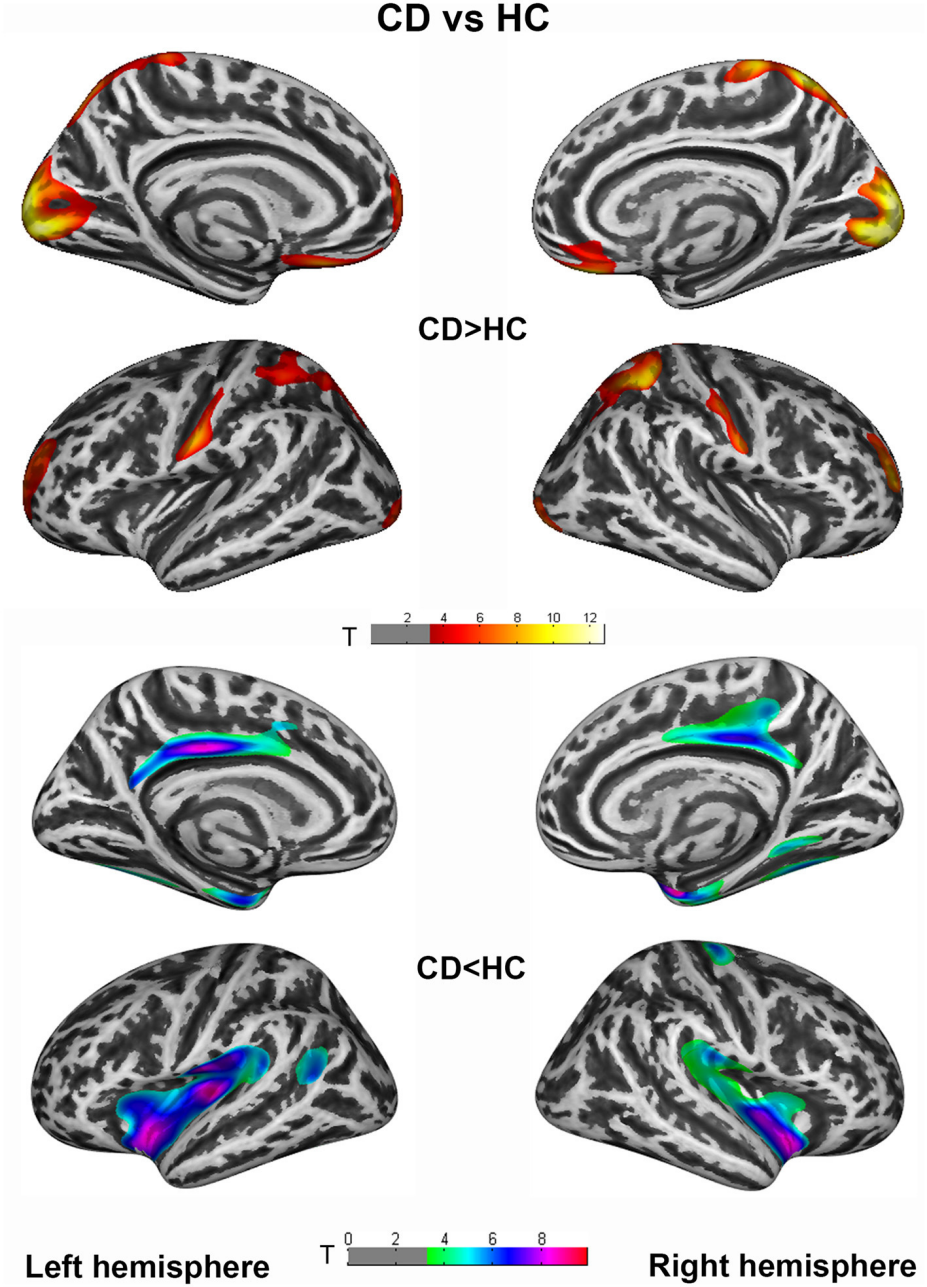
Cortical thickness (CT) differences between cervical dystonia (CD) and healthy controls (HC) group. An independent 2-sample *t*-test was applied to generate statistical maps, presented on inflated surfaces. Results are shown at *P* < 0.005 at cluster level, corrected for multiple comparisons with family wise error (FWE).

**Figure 2:**
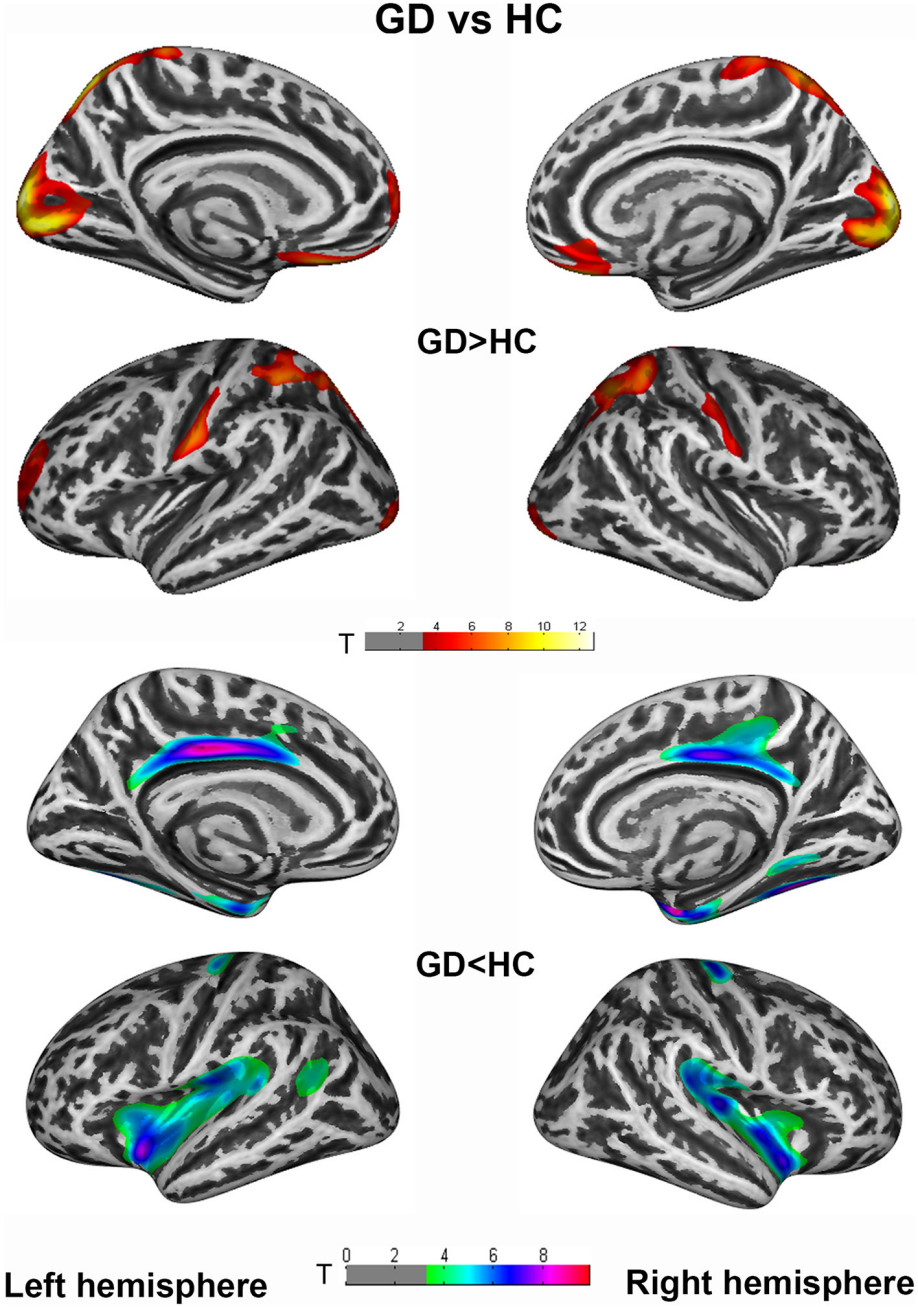
Cortical thickness (CT) differences between generalized dystonia (GD) and healthy controls (HC) group. An independent 2-sample *t*-test was applied to generate statistical maps, presented on inflated surfaces. Results are shown at *P* < 0.005 at cluster level, corrected for multiple comparisons with Family Wise Error (FWE).

Vertex-wise analysis demonstrated significant CT differences of patients in wide-spread cortical regions compared to HCs (*P*_FWE_ <0.005 at cluster level). CD patients relative to HC showed increased CT of bilateral postcentral, superiorparietal, superiorfrontal/rostralmiddlefrontal, occipital gyrus etc., and decreased CT of bilateral cingulate, insula, entorhinal, fusiform, and right precental gyrus. Highly similar increased CT clusters were also displayed when comparing GD to HC. Apart from the regions mentioned above, decreased CT clusters of GD were displayed in bilateral precentral gyrus (Figures 1, 2). However, no significant result survived in comparison between CD and GD (detailed vertices' coordinates, T value, clusters' sizes, overlapped atlas regions, etc. are displayed in Supplementary Table 1).

### Subcortical Volumes and Shape Analysis

**Figure 3:**
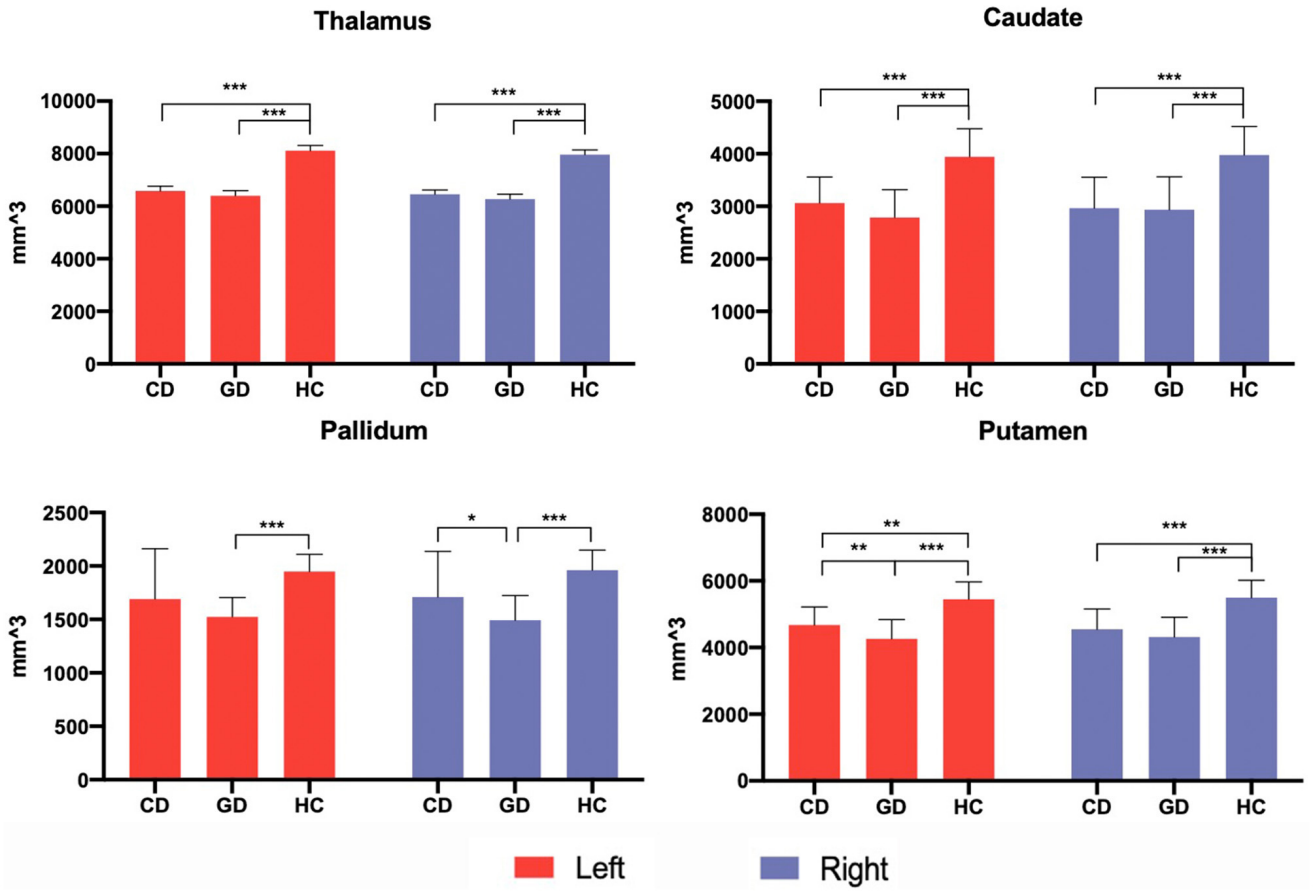
Subcortical volumetric analysis in patients and control groups. Subcortical volumes differences among three groups were analyzed *via* an independent 2-sample *t*-test, with age, gender, and total intracranial volume (TIV) adjusted. The significant threshold level was set at *P* < 0.005 after multiple comparisons were corrected. The trend-level was *P* < 0.05. *P < 0.05; **P < 0.005; ***P < 0.001.

Patients relative to HC showed a general decrease of subcortical structures' volumes. Compared to HC, CD patients showed decreased volumes of the thalamus, caudate, and putamen bilaterally. GD relative to HC showed decreased volume of thalamus, caudate, pallidum, and putamen bilaterally. GD relative to CD showed a decreased volume of right pallidum (*P* < 0.005) and a trend of decreased volume in the left putamen (*P* < 0.05) (Figure 3).

**Figure 4:**
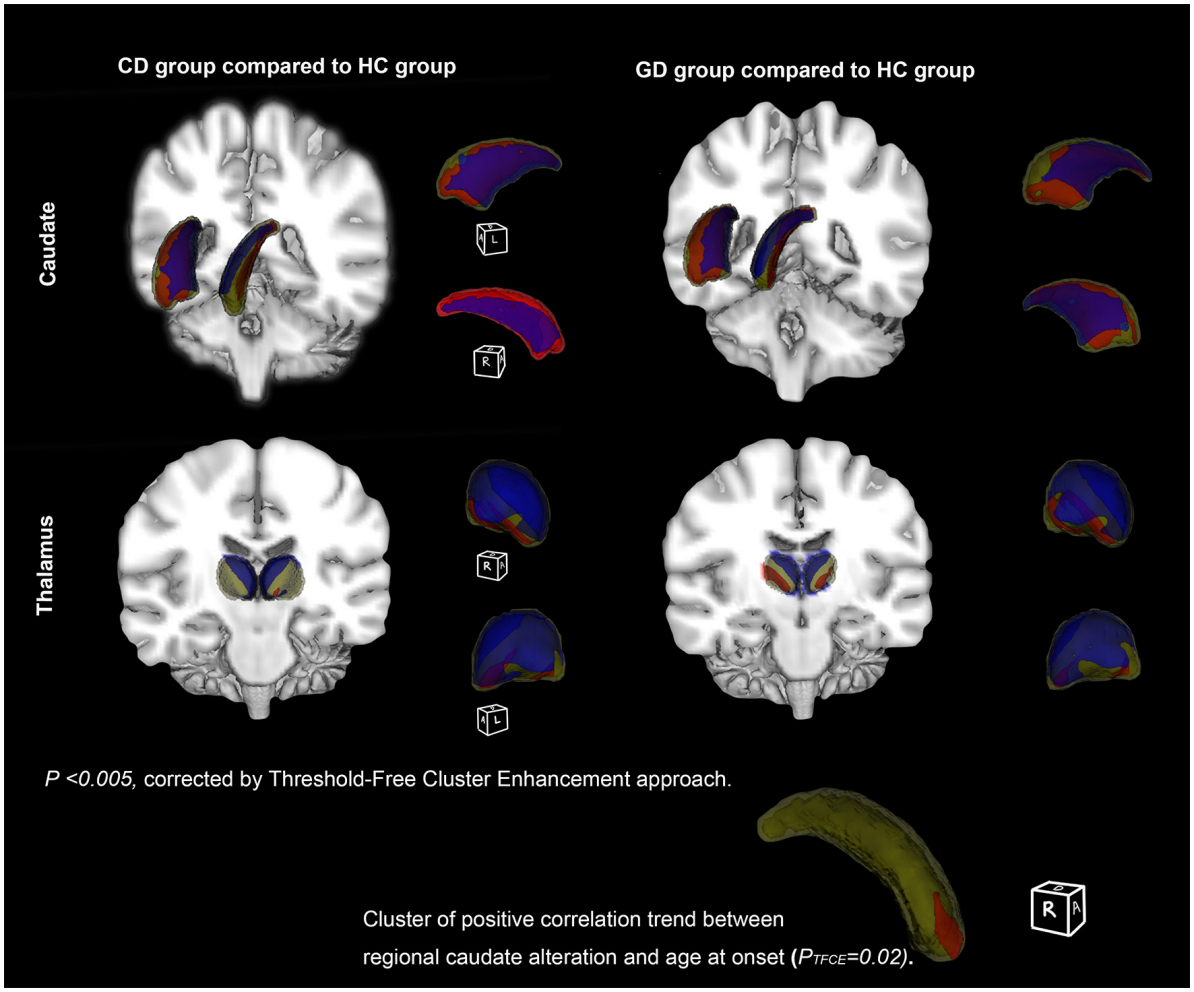
Subcortical structures' shape differences between patient and healthy controls (HC). Vertex-wise comparisons of subcortical structures' shapes between dystonia patients and HC were displayed on fslview. The first row **(left)** showed structures with shape alterations between cervical dystonia (CD) and HC patients (CD vs. HC), while the second row **(right)** showed the differences between generalized dystonia (GD) and HC (GD vs. HC). Clusters presented are corrected for multiple testing using Threshold-Free Cluster Enhancement (TFCE) approach (permutation times = 5,000, *P*_FWE_ <0.005). Significant inward bending cluster was presented in blue, and outward bending cluster in red. In GD, a trend of positive correlation was found between lateral border displacement and age at onset in the anterior and dorsal portion (head) of the right caudate (*P* = 0.02).

The result of vertex-wise shape analysis further revealed significant regional volume alterations of bilateral thalamus and caudate in both CD and GD compared to HC (Figure 4). In both the hemispheres, caudates of patients compared to HC demonstrated inward bending of medial region and outward bending of the lateral region from head to tail, reflecting a general lateral displacement. In the thalamus, the inward bending occurred mainly at medial and dorsal regions, while the outward bending occurred at a small portion of the ventral region (*P*_*TFCE*_ <0.005).

### Correlation Between MRI Features and Clinical Characteristics

Significant negative correlations were only found between CT (ROIs) of the left superiorparietal gyrus, right rostralmiddlefrontal, and TWSTRS pain scores in the CD group (*P* < 0.0025). Under a looser threshold (*P* < 0.05 without correction), trends of negative correlations between their TWSTRS scores and CT were enlarged to bilateral rostralmiddlefrontal, postcentral, left supramarginal, superiorparietal, and precentral gyrus. Negative correlations were also found between disease duration, onset age, and left transversetemporal gyrus, while a positive correlation presented between onset age and the right supramarginal gyrus. In the GD group, we found trends of negative correlations between patients' BFMDRS scores and the left supramarginal, and positive correlation between BFMDRS disability scores and the right inferiorparietal gyrus (Detailed information of correlation matrix, corresponding coefficients, and *P-*values are presented in Supplementary Figure 1; Supplementary Table 2).

**Figure 5:**
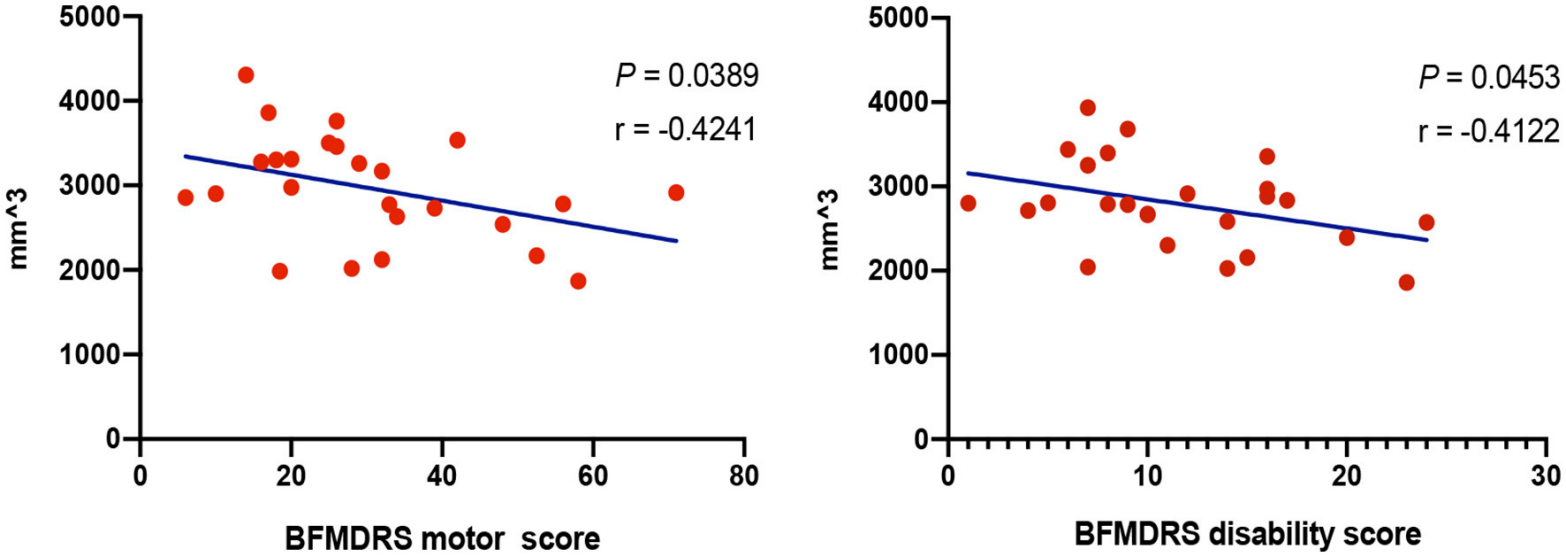
Correlations between volumes of subcortical structures and clinical characteristics. Correlation analysis demonstrated trends of negative correlations with Burke-Fahn-Marsden Dystonia Rating Scale (BFMDRS) disability and motor scores in generalized dystonia (GD), respectively (*P* < 0.05), with age, gender, and total intracranial volume adjusted.

No significant correlation was found in volumetric and shape analysis. However, volumes of left and right caudate, respectively, showed trends of negative correlations with BFMDRS disability and motor scores in GD (*P* < 0.05) (Figure 5). Besides, the outward bending of the dorsal and anterior (head) part of right caudate was discovered of the positive correlation trend with onset age of GD in further shape analysis (*P*_*TFCE*_ = 0.02) (Figure 4).

## Discussion

In this study, we applied three different imaging approaches including SBM, volumetric, and shape analysis, revealing morphometric abnormalities in wide-spread cortical and subcortical regions. The results supported a “network dysfunction” in the pathophysiology of idiopathic dystonia and implied how the affected brain structures might relate to the clinical characteristics.

Cortical abnormalities in the study mainly involved pre/postcentral, rostralmiddlefrontal, lateraloccipital, cingulate, insula gyrus, etc. Those regions with significant differences are responsible for more than movement controlling, plenty of which dominate non-motor processing encompassing sensory, verbal, visual, motor imagination, and cognition (Fiorio et al., 2011; Castrop et al., 2012). In accordance with the imaging findings, non-motor manifestations, like sensory dysfunction (Naumann et al., 2000) commonly occur in patients with dystonia for the evidence of observed increased spatial/temporal discrimination threshold (Tinazzi et al., 1999) and sensory tricks used to neutralize abnormal postures. In addition, working memory, processing speed, and part of social cognition have been presented mildly/moderately defected in primary or secondary dystonia (Jahanshahi et al., 2011; Owen et al., 2015; Coenen et al., 2018). The regions are also largely overlapped with those from a previous study (Simonyan and Ludlow, 2012), which explored CT changes in another form of focal dystonia and demonstrated increased CT in sensorimotor, inferior frontal, superior/middle temporal, and supramarginal gyrus. Nevertheless, the current literature referring to CT abnormalities in dystonia is still inadequate. VBM is a classic and prevalent technique for morphometric analysis by measuring whole brain GMV, and one meta-analysis up to now congregated VBM studies using the anatomic likelihood ratio estimation (ALE) method. The results showed an increased GMV in the primary sensorimotor cortex and a decreased GMV in the thalamus and putamen in patients with primary focal dystonia (Zheng et al., 2012). However, one limitation of VBM is that the results are affected by cortical surface area and total brain volume. Comparatively, SBM is based on surface measurement, such as distance between the white (gray-to-white interface) and pial (gray-to-CSF interface) surfaces for estimating CT, so it is complementary to volumetric analysis, more sensitive, and better for describing cortical column abnormalities. Furthermore, these structural alterations of cortices are often accompanied with metabolic changes in similar brain regions. In positron emission tomography studies, the abnormalities in regional cerebral blood flow (rCBF) measured with [15O]-H2O were mainly cortically localized, including decreased rCBF in the primary motor cortex and increased rCBF in frontal and parietal association areas (Naumann et al., 2000). With the basis of imaging and physiological evidence, these morphometric changes could be interpreted as consequences of maladaptive neuronal plasticity, which eventually lead to cortical network reshaping.

It is striking to notice that the alteration patterns in the two patient groups are highly similar in the face of robust statistical correction (*P*_FWE_ <0.005). From one aspect, it could explain the shared clinical manifestations of disturbed sensorimotor reflection, impaired cognition, and motor imagination in various forms of dystonia. More importantly, updated physiological studies have also indicated a large amount of mechanism overlaps in focal and generalized dystonia, including a similar pattern of short-interval intracortical inhibition measurements and range of temporal discrimination thresholds (Aglioti et al., 2003; Defazio et al., 2007; Fiorio et al., 2007). In this case, the similarity of structural altering patterns could strengthen the theory of common pathophysiological mechanisms underlying CD and GD, or even other forms of idiopathic dystonia. Though CT comparison between CD and GD revealed no significant difference, the exclusively decreased CT of right precentral gyrus in GD relative to HC may indicate trends of more atrophic cortex in the primary motor region with wider body regions involved in patients.

In CD, trends of negative correlations were found between TWSTRS scores and CT in the left precentral supramarginal, superiorparietal, bilateral rostralmiddlefrontal, and postcentral gyrus, indicating that progression of dystonia symptoms could be caused by cortical thinning. Negative correlations in CD have been previously found between disease severity and CT of right precentral and left medial orbitofrontal gyrus (Vilany et al., 2017), and a GMV reduction in somatosensory cortex was demonstrated in a longitudinal study of a 5-year follow-up (Pantano et al., 2011), which reinforced the clinical associations of those critical cortical regions. In GD group, on the contrary, such association was much less evident and only found in the left supramarginal gyrus. On one side, neck is the only affected body region in CD, leading to highly comparable rating scores between patients, whereas in GD, symptom patterns and distributions are highly variable that could partially affect the correlation results. More importantly, we cannot exclude the possibility that cortical regions participate more in the pathophysiology of CD than GD.

At the subcortical level, BG and thalamus were selected in further volumetric and shape analysis. BG, including the striatum (caudate, putamen, and nucleus accumbens), subthalamic nucleus (STN), pallidum, and substantia nigra are traditionally thought to play the causative role in selection and inhibition of competing actions, analogous to a “braking system” (Mink, 2018). One prevailing hypothesis has been postulated that the imbalanced direct and indirect BG pathway leads to a net reduction in the level of inhibitory pallidal output to the thalamus and disinhibition of the motor cortex, which causes involuntary movements in dystonia (Simonyan et al., 2017). Results from the analysis demonstrated that volumes of the bilateral thalamus, caudate, and putamen in both the patient groups were decreased. The bilateral pallidum volume was additionally decreased in patients with GD, revealing the more pronounced subcortical abnormalities in GD. Volumetric reduction indicates a loss of neurons/connections, and may attribute to the impairment of dopamine function and decreased binding of D2 in the striatum and thalamus (Karimi and Perlmutter, 2015), but we were unable to directly determine which cellular/ axonal populations are altered in the nucleus and whether these changes are primary. As a relay center subserving sensory, motor, and other non-motor mechanisms, decreased volume of the thalamus seems to be persistent in VBM studies (Obermann et al., 2007; Waugh et al., 2016). Results of our shape analysis further locate the territory of surface alterations in the thalamus, showing a significant inward bending/atrophy of its medial and dorsal portion, areas that largely contain motor thalamus. Caudate is another crucial nucleus in our findings with its shape deformities and clinical associations. Negative correlations in caudate volumes were found with the disease duration and severity in GD, indicating increasing loss of caudate neurons during disease progression. Last, the atrophy of the right caudate head was located, which implied to an earlier age of onset in patients with GD. Region of the caudate head has been proved strongly connected with medial frontal pole and involved in working memory and executive functioning (Driscoll et al., 2020). Consistently, in another study including CD, a GMV reduction in the caudate head was also reported (Pantano et al., 2011). Our supplementary imaging findings highlighted the role of caudate in idiopathic dystonia.

This is the first study to explore cortical and subcortical structures in both focal and generalized dystonia, which provided supplementary evidence and strengthened the postulated model that dystonia arises from a deranged network interaction among several neural structures. Some notable questions exist yet, and one of them would be the inconsistencies in the change in directions of morphometric measurements across various studies. GMV of prefrontal, inferior parietal, and superior temporal gyrus have been reported to be either increased or decreased in the focal dystonia (Draganski et al., 2003; Egger et al., 2007; de Vries et al., 2008), and some other studies presented no abnormalities in the subcortical structures (Vilany et al., 2017). It is hard to clearly explain these conflicting results, when especially in identical forms of dystonia. With improving accuracy and reliability of imaging algorithms, we assume that the alterations' discrepancies could be formed under the dynamic remodeling of cortical and subcortical organization, where neuron loss and compensatory formation of new synaptic connections coexist (Chklovskii, 2004; Sur and Rubenstein, 2005). Thus, a longitude study of tracking patients for years would help to illuminate on how progression of the disease may affect the dynamic alterations of brain structures.

How would the imaging assessment be potentially applied to the clinic? The diagnosis of dystonia is mainly dependent on clinical manifestations. With the “shared imaging features” in idiopathic dystonia, additional imaging clarification might help to recognize or exclude some other dystonic-like movement disorders, which are controversial. Moreover, imaging measurements that are related to clinical characteristics could serve as critical features for predicting the benefits from treatments.

The study embraces several limitations: due to methodological limitations, some subcortical structures were not evaluated. For instance, STN connects cortical regions and striatum, being an effective neuromodulation target in dystonia as well, but the structure is tiny and unable to be segmented automatically. In addition, cerebellum was not discussed in our study either, though increasing evidence have pointed to the candidate of cerebello-thalamo-cortical fiber tracts in the pathophysiology of dystonia with DYT1/DYT6 mutations (Argyelan et al., 2009; Neychev et al., 2011). In terms of the analysis tools, CAT12 and FreeSurfer (http://surfer.nmr.mgh.harvard.edu/) are two software utilizing surface-based approaches that are available for CT estimation currently. FreeSurfer is an established software with high accuracy of measurement, but it was not applied in our study because it is a highly time-consuming segmentation process. CAT12 offers a fast process approach, a graphical user interface, and at the same time has a comparable accuracy with Freesurfer (Seiger et al., 2018; Velázquez et al., 2021). Nevertheless, comparing measurements with two different software would be appropriate for further improving the reliability of results.

As a final remark, the current results in our study cannot fully differentiate between causative and compensatory deficits in the disease. Future studies conducted with larger sample size, more detailed classification, and multiple imaging techniques would be necessary for linking different findings together and drawing more reliable conclusions.

## Conclusions

The study shows CT abnormalities of pre/postcentral, frontal, temporal, supramarginal, cingulate, insula gyrus, etc., as well as subcortical volume and shape alterations in idiopathic dystonia, indicating a network defect and reshaping in disease progression. Regarding highly overlapped brain regions in CD and GD with significant differences, and consistent results in previous studies, we assumed that these abnormalities serve as shared features in the mechanism of idiopathic dystonia, which might help to differentiate similar diseases. Secondly, subcortical volumes were broadly decreased in patients, accompanied by regional shape alterations of bilateral thalamus and caudate. In analyses of clinical associations, cortical thinning of pre/postcentral, frontal, parietal, and supramarginal gyrus were correlated to severe symptoms in CD, whilst the volume reduction and shape atrophy of caudate were correlated to severer symptoms and earlier age of onset in GD at a trend level. These results supported the causal roles of the above cortical regions and caudate in the pathophysiology of dystonia.

## Data Availability Statement

The raw data supporting the conclusions of this article will be made available by the authors, without undue reservation.

## Ethics Statement

The studies involving human participants were reviewed and approved by Ruijin Hospital, Shanghai Jiao Tong University School of Medicine. The patients/participants provided their written informed consent to participate in this study.

## Author Contributions

YuW: conceptualization and writing-original draft. QD: methodology and software. TW and HL: data curation and preparation. TW: visualization and investigation. YiW and DL: supervision. YP and BS: writing-reviewing and editing. All authors contributed to the article and approved the submitted version.

## Funding

This study was supported by the Medical and Engineering Foundation of Shanghai Jiao Tong University (Grant Number WF540162605).

## Conflict of Interest

The authors declare that the research was conducted in the absence of any commercial or financial relationships that could be construed as a potential conflict of interest.

## Publisher's Note

All claims expressed in this article are solely those of the authors and do not necessarily represent those of their affiliated organizations, or those of the publisher, the editors and the reviewers. Any product that may be evaluated in this article, or claim that may be made by its manufacturer, is not guaranteed or endorsed by the publisher.
