# Supplementary material for: Cortical and Subcortical Structural Abnormalities in Patients With Idiopathic Cervical and Generalized Dystonia
Source: Front Neuroimaging. 2022 Mar 31;1:807850. doi: 10.3389/fnimg.2022.807850 (PMC10406292; doi:10.3389/fnimg.2022.807850)
Supplement: Supplementary file 1 [file Data_Sheet_1.docx]

Supplementary Material

# Supplementary Figures

**Supplementary Figure 1. Correlation plots between mean cortical thickness of regions of interest and clinical characteristics in dystonia patients.**

**
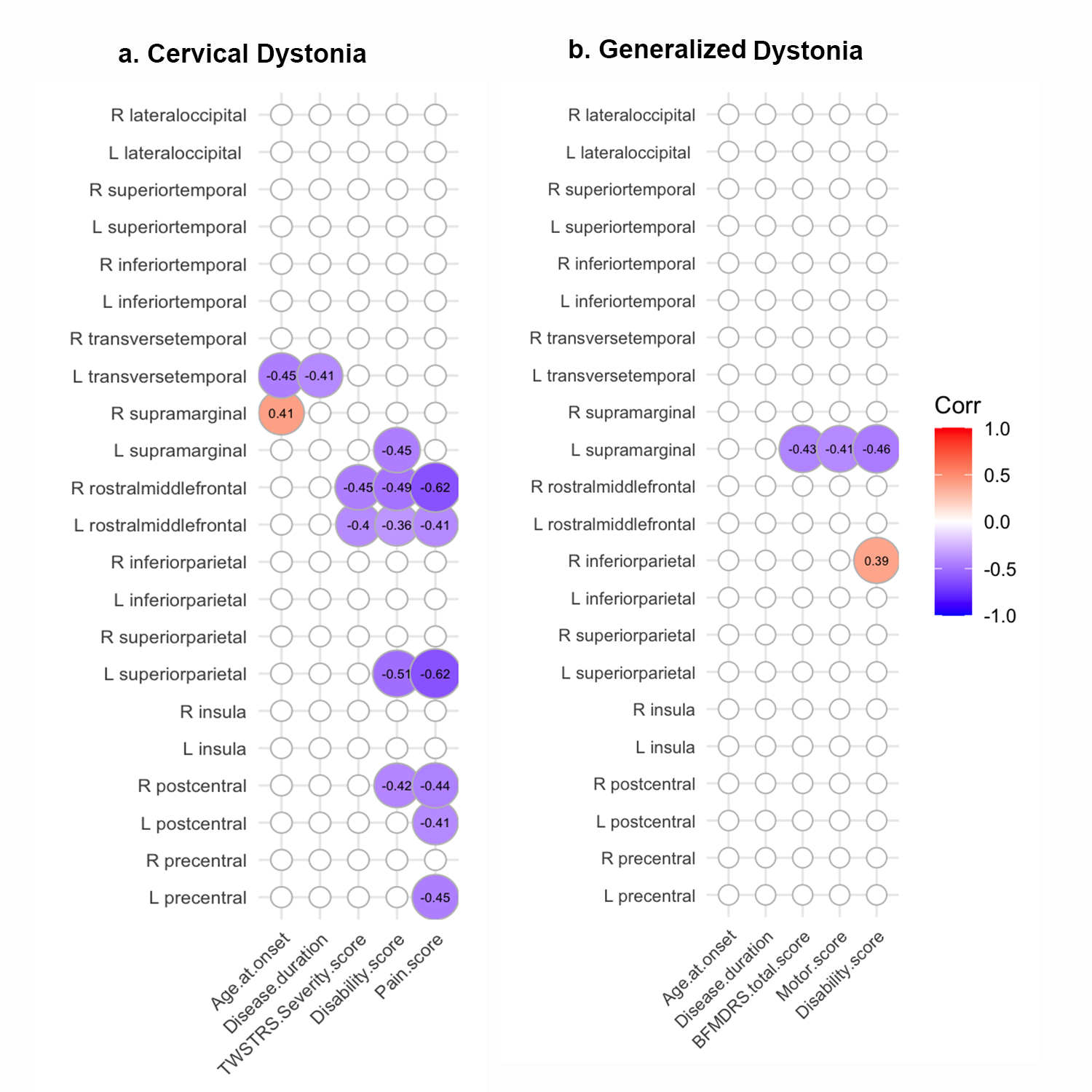
**

The mean values were extracted from regions of interest (ROIs) which mainly overlapped clusters with significant differences from previous vertex-wise comparisons, based on Desikan-Killiany Atlas (DK40). Cortical thickness of ROIs were correlated with patients’ clinical characteristics including age at onset, disease duration, and scores of rating scales. Significant statistical threshold was set at *P<*0.0025 after multiple comparisons correction, and Pearson correlation coefficients marked in the circles were present under a threshold of *P<*0.05 (trend level). BFMDRS, Burke-Fahn-Marsden Dystonia Rating Scale; TWSTRS, Toronto Western Spasmodic Torticollis Rating Scale.

# Supplementary Tables

**Supplementary Table 1. Cortical thickness alterations in dystonia patients.**

a. Increased cortical thickness in CD patients compared to HC.

|  |  |  | MNI coordinates  (maximum vertex, mm) | | |  |  |
| --- | --- | --- | --- | --- | --- | --- | --- |
| Cluster no. | Cluster size (vertices) | Cluster-wise P | x | y | z | T-Value (maximum vertex) | Overlap of atlas region |
| 1 | 1000 | <0.001 | -9 | -49 | 32 | 11.55 | L lingual |
| 2 | 630 | <0.001 | -10 | -59 | 45 | 11.04 | L precuneus |
| 3 | 1397 | <0.001 | -57 | 25 | 65 | 8.30 | L postcentral |
| 4 | 1527 | 0.001 | -11 | 58 | 21 | 7.75 | L pericalcarine, lateraloccipital |
| 5 | 4275 | <0.001 | -19 | -36 | 88 | 7.55 | L superiorparietal |
| 6 | 782 | <0.001 | -6 | 82 | 49 | 6.63 | L superiorfrontal |
| 6 | 1779 | <0.001 | -21 | 86 | 47 | 6.97 | L rostralmiddlefrontal |
| 7 | 891 | <0.001 | 4 | -51 | 47 | 9.44 | R pericalcarine, lateraloccipital |
| 8 | 891 | <0.001 | 1 | -51 | 39 | 9.29 | R lingual |
| 9 | 1006 | <0.001 | 7 | -22 | 93 | 9.09 | R precuneus |
| 10 | 4528 | <0.001 | 30 | -13 | 87 | 7.42 | R superiorparietal |
| 11 | 498 | <0.001 | 7 | 64 | 19 | 8.23 | R laterlorbitofrontal |
| 12 | 1007 | 0.001 | 21 | 76 | 40 | 7.13 | R rostralmiddlefrontal |
| 13 | 1580 | <0.001 | 56 | 23 | 49 | 6.10 | R postcentral |

b. Decreased cortical thickness in CD patients compared to HC.

|  |  |  | MNI coordinates  (maximum vertex, mm) | | |  |  |
| --- | --- | --- | --- | --- | --- | --- | --- |
| Cluster no. | Cluster size (vertices) | Cluster-wise P | x | y | z | T-Value (maximum vertex) | Overlap of atlas region |
| 1 | 4613 | <0.001 | -38 | 8 | 45 | 8.29 | L insula, transversetemporal, superiortemporal |
| 2 | 1663 | <0.001 | -3 | 9 | 67 | 7.93 | L posteriorcingulate, isthmuscingulate |
| 3 | 848 | 0.002 | -43 | -23 | 56 | 5.78 | L inferiorparietal |
| 4 | 925 | 0.001 | -30 | 27 | 9 | 6.25 | L entorhinal |
| 5 | 776 | 0.006 | -36 | -26 | 31 | 5.17 | L fusiform |
| 6 | 883 | <0.001 | 16 | 31 | 10 | 9.18 | R entorhinal |
| 7 | 3729 | <0.001 | 33 | 31 | 20 | 8.82 | R insula, transversetemporal, superiortemporal |
| 8 | 1867 | <0.001 | 3 | 12 | 68 | 7.30 | R posteriorcingulate, isthmuscingulate |
| 9 | 1172 | <0.001 | 20 | 3 | 95 | 6.41 | R precentral |
| 10 | 1072 | 0.001 | 27 | -9 | 18 | 6.14 | R fusiform |

c. Increased cortical thickness in GD patients compared to HC.

|  |  |  | MNI coordinates  (maximum vertex, mm) | | |  |  |
| --- | --- | --- | --- | --- | --- | --- | --- |
| Cluster no. | Cluster size (vertices) | Cluster-wise P | x | y | z | T-Value (maximum vertex) | Overlap of atlas region |
| 1 | 1497 | <0.001 | -11 | -50 | 19 | 9.64 | L pericalcarine, lateraloccipital |
| 2 | 981 | <0.001 | -16 | -41 | 13 | 8.77 | L lingual |
| 3 | 4304 | <0.001 | -18 | -37 | 70 | 7.90 | L superiorparietal |
| 4 | 1009 | 0.001 | -15 | 70 | 9 | 6.94 | L medialorbitofrontal |
| 5 | 1363 | <0.001 | -63 | 29 | 56 | 6.05 | L postcentral |
| 6 | 741 | <0.001 | -6 | 95 | 35 | 4.82 | L superiorfrontal |
| 7 | 1482 | <0.001 | -25 | 88 | 50 | 4.78 | L rostralmiddlefrontal |
| 8 | 890 | <0.001 | 5 | -44 | 32 | 10.86 | R pericalcarine, lateraloccipital |
| 9 | 890 | <0.001 | 3 | -29 | 15 | 8.35 | R lingual |
| 10 | 1062 | <0.001 | 8 | 78 | 2 | 9.54 | R medialorbitofrontal |
| 11 | 4529 | <0.001 | 21 | -13 | 69 | 7.50 | R superiorparietal |
| 12 | 1500 | <0.001 | 51 | 20 | 60 | 5.22 | R postcentral |
| 13 | 1078 | <0.001 | 9 | -18 | 79 | 7.63 | L precuneus |

d. Decreased cortical thickness in GD patients compared to HC.

|  |  |  | MNI coordinates  (maximum vertex, mm) | | |  |  |
| --- | --- | --- | --- | --- | --- | --- | --- |
| Cluster no. | Cluster size (vertices) | Cluster-wise P | x | y | z | T-Value (maximum vertex) | Overlap of atlas region |
| 1 | 1660 | <0.001 | -5 | 21 | 51 | 8.95 | L posteriorcingulate, isthmuscingulate |
| 2 | 4483 | <0.001 | -38 | 41 | 18 | 7.92 | L insula, transversetemporal, superiortemporal |
| 3 | 1834 | <0.001 | -36 | 15 | 37 | 5.8 | L supramarginal |
| 4 | 934 | 0.001 | -32 | 36 | -6 | 6.54 | L entorhinal |
| 5 | 891 | 0.003 | -27 | 11 | 85 | 5.79 | L precentral |
| 6 | 832 | 0.002 | -50 | -14 | 37 | 4.57 | L inferiorparietal |
| 7 | 8729 | <0.001 | 24 | 45 | -7 | 9.36 | R entorhinal |
| 8 | 1194 | <0.001 | 32 | -2 | 0 | 9.01 | R fusiform |
| 9 | 1869 | <0.001 | 3 | 22 | 54 | 7.97 | R posteriorcingulate, isthmuscingulate |
| 10 | 1172 | <0.001 | 25 | 13 | 81 | 7.06 | R precentral |
| 11 | 3686 | <0.001 | 35 | 41 | 11 | 7.44 | R insular, transversetemporal, superiortemporal |
| 12 | 2037 | <0.001 | 34 | 12 | 26 | 7.40 | R supramarginal |

Results of maximum vertices coordinates, T values, cluster sizes, and overlapped atlas regions. All results are considered significant at *P*<0.005 at cluster level (3 group comparisons for each hemisphere, 0.005 < 0.05/6), corrected for multiple comparisons with Family Wise Error (FWE). Coordinates are presented on standard MNI152 template (1.5mm*1.5mm*1.5mm). The clusters are listed in a random order. CD, cervical dystonia; GD, generalized dystonia; HC, healthy controls.

**Supplementary Table 2. Correlations between mean cortical thickness of regions of interest and clinical characteristics in dystonia patients (*P-*value matrix).**

a. Cervical dystonia

|  | Age at onset | Disease duration | TWSTRS Severity score | Disability score | Pain score |
| --- | --- | --- | --- | --- | --- |
| L precentral | 0.330 | 0.197 | 0.958 | 0.421 | 0.013 |
| R precentral | 0.212 | 0.086 | 0.942 | 0.400 | 0.118 |
| L postcentral | 0.080 | 0.214 | 0.683 | 0.672 | 0.025 |
| R postcentral | 0.596 | 0.233 | 0.186 | 0.022 | 0.014 |
| L insula | 0.224 | 0.571 | 0.203 | 0.265 | 0.642 |
| R insula | 0.262 | 0.069 | 0.283 | 0.338 | 0.797 |
| L superiorparietal | 0.733 | 0.347 | 0.050 | 0.004 | <0.001 |
| R superiorparietal | 0.614 | 0.434 | 0.386 | 0.124 | 0.227 |
| L inferiorparietal | 0.237 | 0.438 | 0.125 | 0.102 | 0.473 |
| R inferiorparietal | 0.533 | 0.719 | 0.153 | 0.245 | 0.803 |
| L rostralmiddlefrontal | 0.359 | 0.903 | 0.027 | 0.049 | 0.026 |
| R rostralmiddlefrontal | 0.637 | 0.687 | 0.012 | 0.006 | 0.001 |
| L supramarginal | 0.174 | 0.780 | 0.073 | 0.012 | 0.198 |
| R supramarginal | 0.025 | 0.756 | 0.056 | 0.248 | 0.535 |
| L transversetemporal | 0.013 | 0.024 | 0.792 | 0.474 | 0.344 |
| R transversetemporal | 0.501 | 0.154 | 0.724 | 0.612 | 0.640 |
| L inferiortemporal | 0.976 | 0.339 | 0.773 | 0.519 | 0.806 |
| R inferiortemporal | 0.989 | 0.824 | 0.958 | 0.913 | 0.268 |
| L superiortemporal | 0.908 | 0.567 | 0.200 | 0.135 | 0.355 |
| R superiortemporal | 0.905 | 0.889 | 0.781 | 0.787 | 0.990 |
| L lateraloccipital | 0.570 | 0.892 | 0.167 | 0.166 | 0.074 |
| R lateraloccipital | 0.244 | 0.626 | 0.754 | 0.890 | 0.933 |

b. Generalized dystonia

|  | Age at onset | Disease duration | BFMDRS total score | Motor score | Disability score |
| --- | --- | --- | --- | --- | --- |
| L precentral | 0.553 | 0.781 | 0.681 | 0.587 | 0.769 |
| R precentral | 0.517 | 0.919 | 0.337 | 0.279 | 0.764 |
| L postcentral | 0.100 | 0.949 | 0.253 | 0.204 | 0.508 |
| R postcentral | 0.313 | 0.569 | 0.704 | 0.576 | 0.836 |
| L insula | 0.585 | 0.970 | 0.918 | 0.971 | 0.730 |
| R insula | 0.084 | 0.690 | 0.100 | 0.062 | 0.166 |
| L superiorparietal | 0.869 | 0.886 | 0.219 | 0.213 | 0.130 |
| R superiorparietal | 0.638 | 0.573 | 0.452 | 0.488 | 0.416 |
| L inferiorparietal | 0.810 | 0.429 | 0.654 | 0.517 | 0.772 |
| R inferiorparietal | 0.843 | 0.766 | 0.147 | 0.157 | 0.049 |
| L rostralmiddlefrontal | 0.288 | 0.153 | 0.773 | 0.800 | 0.641 |
| R rostralmiddlefrontal | 0.287 | 0.179 | 0.911 | 0.925 | 0.770 |
| L supramarginal | 0.472 | 0.633 | 0.026 | 0.037 | 0.019 |
| R supramarginal | 0.244 | 0.866 | 0.958 | 0.972 | 0.469 |
| L transversetemporal | 0.360 | 0.887 | 0.651 | 0.662 | 0.785 |
| R transversetemporal | 0.189 | 0.946 | 0.720 | 0.628 | 0.715 |
| L inferiortemporal | 0.370 | 0.237 | 0.998 | 0.990 | 0.861 |
| R inferiortemporal | 0.471 | 0.559 | 0.467 | 0.473 | 0.854 |
| L superiortemporal | 0.677 | 0.286 | 0.209 | 0.268 | 0.093 |
| R superiortemporal | 0.542 | 0.968 | 0.897 | 0.953 | 0.567 |
| L lateraloccipital | 0.172 | 0.649 | 0.268 | 0.287 | 0.140 |
| R lateraloccipital | 0.197 | 0.574 | 0.272 | 0.277 | 0.159 |

Statistical *P*-value results in Pearson correlation analysis of cortical thickness and clinical characteristics.
